# History of chickenpox in glioma risk: a report from the glioma international case–control study (GICC)

**DOI:** 10.1002/cam4.682

**Published:** 2016-03-13

**Authors:** E. Susan Amirian, Michael E. Scheurer, Renke Zhou, Margaret R. Wrensch, Georgina N. Armstrong, Daniel Lachance, Sara H. Olson, Ching C. Lau, Elizabeth B. Claus, Jill S. Barnholtz‐Sloan, Dora Il'yasova, Joellen Schildkraut, Francis Ali‐Osman, Siegal Sadetzki, Robert B. Jenkins, Jonine L. Bernstein, Ryan T. Merrell, Faith G. Davis, Rose Lai, Sanjay Shete, Christopher I. Amos, Beatrice S. Melin, Melissa L. Bondy

**Affiliations:** ^1^ Department of PediatricsDivision of Hematology‐OncologyDan L. Duncan Cancer CenterBaylor College of MedicineHoustonTexas; ^2^ Department of Neurological SurgeryUniversity of CaliforniaSan FranciscoCalifornia; ^3^ Department of NeurologyMayo Clinic Comprehensive Cancer CenterMayo ClinicRochesterMinnesota; ^4^ Department of Epidemiology and BiostatisticsMemorial Sloan‐Kettering Cancer CenterNew YorkNew York; ^5^ Department of Epidemiology and Public HealthYale University School of MedicineNew HavenConnecticut; ^6^ Department of NeurosurgeryBrigham and Women's HospitalBostonMassachusetts; ^7^ Case Comprehensive Cancer CenterCase Western Reserve University School of MedicineClevelandOhio; ^8^ Department of Epidemiology and BiostatisticsGeorgia State University School of Public HealthAtlantaGeorgia; ^9^ Department of Community and Family MedicineCancer Control and Prevention ProgramDuke University Medical CenterDurhamNorth Carolina; ^10^ Department of SurgeryDuke University Medical CenterDurhamNorth Carolina; ^11^ Cancer and Radiation Epidemiology UnitGertner InstituteChaim Sheba Medical CenterTel HashomerIsrael; ^12^ Sackler School of MedicineTel‐Aviv UniversityTel‐AvivIsrael; ^13^ Department of Laboratory Medicine and PathologyMayo Clinic Comprehensive Cancer CenterMayo ClinicRochesterMinnesota; ^14^ Department of NeurologyNorthShore University HealthSystemEvanstonIllinois; ^15^ Department of Public Health ServicesUniversity of AlbertaEdmontonAlbertaCanada; ^16^ Departments of Neurology, Neurosurgery, and Preventive MedicineThe University of Southern California Keck School of MedicineLos AngelesCalifornia; ^17^ Department of BiostatisticsThe University of Texas MD Anderson Cancer CenterHoustonTexas; ^18^ Department of Community and Family MedicineDepartment of GeneticsNorris Cotton Cancer CenterGeisel School of Medicine at DartmouthHanoverNew Hampshire; ^19^ Department of Radiation Sciences OncologyUmeå UniversityUmeåSweden

**Keywords:** Brain tumor, chickenpox, glioma, shingles

## Abstract

Varicella zoster virus (VZV) is a neurotropic *α*‐herpesvirus that causes chickenpox and establishes life‐long latency in the cranial nerve and dorsal root ganglia of the host. To date, VZV is the only virus consistently reported to have an inverse association with glioma. The Glioma International Case‐Control Study (GICC) is a large, multisite consortium with data on 4533 cases and 4171 controls collected across five countries. Here, we utilized the GICC data to confirm the previously reported associations between history of chickenpox and glioma risk in one of the largest studies to date on this topic. Using two‐stage random‐effects restricted maximum likelihood modeling, we found that a positive history of chickenpox was associated with a 21% lower glioma risk, adjusting for age and sex (95% confidence intervals (CI): 0.65–0.96). Furthermore, the protective effect of chickenpox was stronger for high‐grade gliomas. Our study provides additional evidence that the observed protective effect of chickenpox against glioma is unlikely to be coincidental. Future studies, including meta‐analyses of the literature and investigations of the potential biological mechanism, are warranted.

## Introduction

Varicella zoster virus (VZV) is a neurotropic *α*‐herpesvirus that causes chickenpox by initially infecting the respiratory mucosa and then progressing into viremia, during which the virus is transported to and replicates in the skin 1. Prior to the licensing of the live attenuated VZV vaccine in the 1990s, chickenpox was an extremely common childhood illness, affecting over 90% of individuals 2, 3. After acute infection, the virus establishes life‐long latency in the cranial nerve and dorsal root ganglia of the host, and may later reactivate in about 10–20% of VZV‐infected individuals, causing shingles. Viral reactivation can also result in other neurological complications, such as encephalitis and myelitis 2, 4.

Because of its neurotropism and its ability to establish decades‐long latency across the neuraxis, 4 VZV is particularly interesting to investigate in relation to gliomagenesis. In fact, of the many viruses previously suspected to be involved in glioma susceptibility (i.e., simian virus 40, BK virus, JC virus, human cytomegalovirus, human herpesvirus‐6) 5, 6, 7, VZV is the only virus consistently reported to have an inverse association with glioma 3. The observed inverse relationship between VZV infection and glioma risk has remained relatively consistent across studies with different VZV exposure assessment methods, such as self‐reported history of chickenpox 8, 9, total anti‐VZV Immunoglobulin G (IgG) levels 9, 10, 11, 12, and levels of antibodies against specific VZV proteins 13. Furthermore, because of its ability to replicate rapidly and lyse malignant glioma cells in vitro, VZV has even been proposed as a novel candidate for glioma virotherapy 14.

The Glioma International Case‐Control Study (GICC) is a large, multisite consortium with data on 4533 cases and 4171 controls collected across five countries 15. The GICC provides an unparalleled opportunity to confirm the previously reported associations between history of chickenpox and glioma in the largest study to date on this topic.

## Materials and Methods

### Study population

Details on the GICC study population and recruitment methods are available elsewhere 15. Briefly, the GICC is an international consortium with 14 recruitment sites: Brigham and Women's Hospital (Boston, MA, USA), Case Western Reserve University (Cleveland, Ohio, USA), Columbia University (New York, NY, USA), Danish Cancer Society Research Centre (Copenhagen, Denmark), The Gertner Institute (Tel Hashomer, Israel), Duke University (Durham, NC), University of Texas MD Anderson Cancer Center (Houston, TX, USA), Memorial Sloan Kettering Cancer Center (New York, NY, USA), Mayo Clinic (Rochester, MN, USA), NorthShore HealthSystem (Chicago, IL, USA), Umeå University (Umeå, Sweden), University of California, San Francisco (San Francisco, CA, USA), University of Southern California (Los Angeles, CA, USA), and The Institute of Cancer Research (London, United Kingdom). All participating institutions received Institutional Review Board (IRB) or ethical board approval for the study, and informed consent was obtained from participants.

Cases were defined as individuals within 18–80 years of age (at diagnosis) who had one of the following types of histologically confirmed, supratentorial, intracranial gliomas: fibrillary astrocytoma (9420/3), protoplasmic astrocytoma (9410/3), gemistocytic astrocytoma (9411/3), oligodendroglioma (9450/3), oligoastrocytoma (9382/3), anaplastic astrocytoma (9401/3), anaplastic oligodendroglioma (9451/3), anaplastic oligoastrocytoma (9382/3), gliosarcoma (9442/3), and glioblastoma (9440/3). All cases were recruited within a year of diagnosis and consented at their clinic visits. All sites started recruiting participants in April 2010.

Controls were 18–80 years of age. Because not all sites were able to recruit controls using the same methods (due to issues related to existing infrastructure and resources), four sites recruited clinic‐based controls, three sites recruited population‐based controls, and seven sites recruited visitors of cancer patients as controls 15.

### Data collection

All 14 sites used a common study protocol and the same risk factor questionnaire. Study coordinators were trained to ensure site‐to‐site homogeneity in data collection practices. Data were stored in a centralized database, and were managed by the lead statistician. More details on our data collection methods have previously been published 15.

The GICC risk factor questionnaire included demographic characteristics, past medical history, and occupational exposure history. Questionnaires were administered through phone and/or in‐person interviews, or through mailed self‐administered forms. Specifically with regard to VZV‐related conditions, participants were asked whether they had ever had any of a list of viral infections, which included chickenpox and shingles. If they answered yes, they were asked their age or what year it was when they had chickenpox or shingles.

### Statistical analysis

The overall GICC analysis plan, as well as details of key sensitivity analyses, are available elsewhere 15. Here, we compared cases and controls on selected characteristics, overall, by study site [not shown], and by tumor grade (high‐grade: WHO Grade IV; lower grade: Grade II and III) among cases. Self‐reported history of chickenpox and self‐reported history of shingles were the exposures of interest.

Site‐specific unadjusted and adjusted odds ratios (ORs), and their corresponding 95% Wald confidence intervals (CIs), were calculated, using unconditional logistic regression. Sites with less than five cases or controls in the exposed or unexposed groups were excluded from the meta‐analyses. To calculate the meta‐analysis ORs (mOR), we utilized both two‐stage random‐effects maximum likelihood and two‐stage random‐effects restricted maximum likelihood (REML) modeling 15. Only final results from the two‐stage REML are presented, as results were very similar using the other method. The *I* ^*2*^ statistic was used for each meta‐regression model to evaluate the proportion of variability in the effect estimates due to heterogeneity, and the *τ* ^*2*^ statistic was calculated to assess the intersite variance. In some stratified analyses, the numbers became too sparse to calculate mORs, and thus pooled ORs (pORs) had to be provided instead.

Age and sex were considered potential confounders (determined a priori) and were adjusted for in all multivariable models, though adjustment for these factors did not meaningfully alter effect estimates. Education, race/ethnicity, allergy status, and cigarette smoking history were evaluated as potential data‐based confounders and were not found to be such, based on a 10% change‐in‐estimate criterion. These variables were therefore not included in the final models. We also stratified our models by age at chickenpox development (<6, 6–9, and >9 years of age), and separately by glioma diagnosis/study enrollment age groups (<40, 40–59, and >59 years of age).

Sensitivity analyses were conducted in which we included and excluded proxy respondents in the final models and compared the results to ensure that there were no meaningful differences between the ORs. Possible patterns or discrepancies in effect estimates between sites with different control types (visitor, clinic‐, or population‐based) or different questionnaire administration methods (in‐person, mailed, or telephone) were also evaluated 15. All analyses were conducted in SAS 9.2 (SAS Institute, Cary, NC) or R version 3.1.2 (Vienna, Austria, http://www.R-project.org).

## Results

Table 1 provides information on selected characteristics of the GICC study population (4533 total cases and 4171 total controls). A table of the study population demographics by site has previously been published 15. The majority of the study population was non‐Hispanic white, but there was a slightly higher preponderance of non‐Hispanic black race/ethnicity among controls. The age distribution was similar among cases and controls, but as expected, high‐grade glioma cases were slightly older.

**Table 1:** Population characteristics by case‐control status and tumor grade: The Glioma International Case‐Control Study (GICC)

	Case	Control	High‐Grade Cases^a^	Lower Grade Cases^a^
	No.(%)	No.(%)	No.(%)	No.(%)
Sex				
Male	2679 (59.1)	2351 (56.37)	1728 (62.29)	916 (54.3)
Female	1854 (40.9)	1820 (43.63)	1046 (37.71)	771 (45.7)
Diagnosis/enrollment age				
18–29 years	308 (6.79)	294 (7.05)	62 (2.24)	228 (13.52)
30–39 years	521 (11.49)	473 (11.34)	108 (3.89)	398 (23.59)
40–49 years	813 (17.94)	680 (16.3)	417 (15.03)	384 (22.76)
50–59 years	1150 (25.37)	1079 (25.87)	796 (28.7)	338 (20.04)
60–69 years	1239 (27.33)	1098 (26.32)	993 (35.8)	238 (14.11)
70–80 years	502 (11.07)	547 (13.11)	398 (14.35)	101 (5.99)
Education^b^				
Less than high school	1127 (27.53)	912 (22.45)	717 (28.55)	392 (25.82)
Some college	1107 (27.05)	1295 (31.88)	653 (26.01)	434 (28.59)
Bachelor's degree	1031 (25.19)	958 (23.58)	600 (23.89)	415 (27.34)
Advanced degree	816 (19.94)	893 (21.98)	535 (21.31)	271 (17.85)
Missing	12 (0.29)	4 (0.1)	6 (0.24)	6 (0.4)
Race/ethnicity				
Non‐Hispanic white	4163 (91.84)	3691 (88.49)	2577 (92.9)	1522 (90.22)
Non‐Hispanic black	71 (1.57)	139 (3.33)	41 (1.48)	26 (1.54)
Asian	84 (1.85)	87 (2.09)	35 (1.26)	48 (2.85)
Hispanic	162 (3.57)	224 (5.37)	93 (3.35)	67 (3.97)
Other	38 (0.84)	26 (0.62)	22 (0.79)	15 (0.89)
Missing	15 (0.33)	4 (0.1)	6 (0.22)	9 (0.53)
Total	4533 (100)	4171 (100)	2774 (100)	1687 (100)

a The sum of the high‐grade and lower grade cases is not equal to the total number of cases because of unclassified cases.

b One site (UK) did not collect education information.

**Figure 1:**
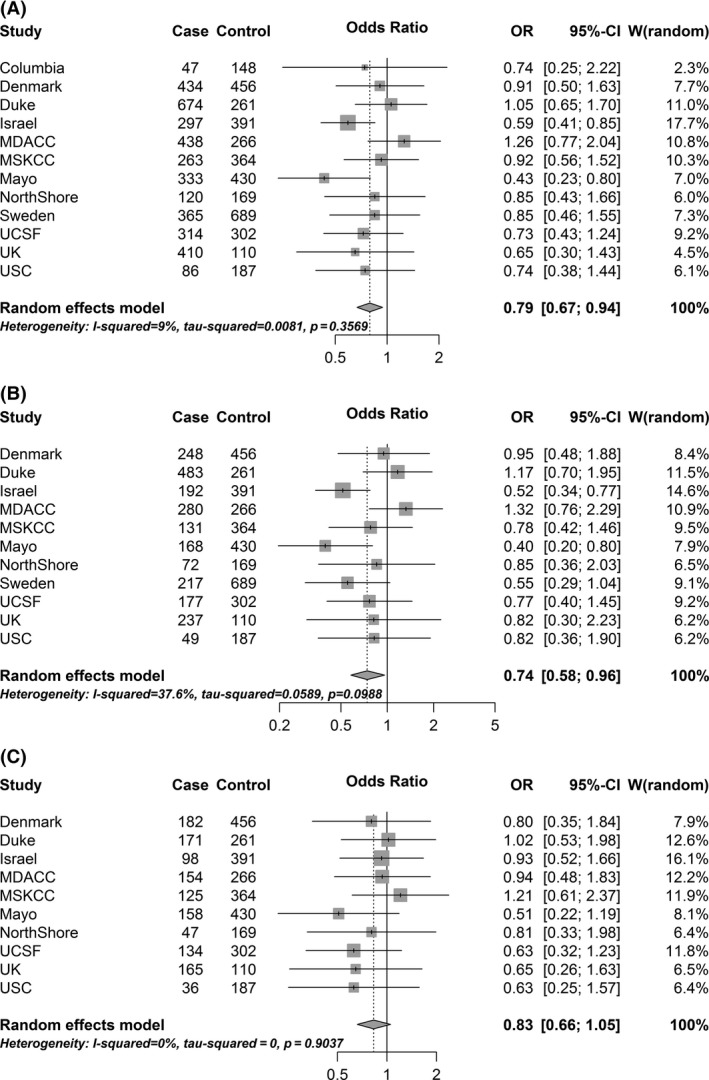
Forest plots for the associations between history of chickenpox and glioma: Findings from the Glioma International Case‐Control Study (GICC). (A) In the overall study population. (B) Among high‐grade glioma. (C) Among lower grade glioma.

Approximately, 79% of cases and 83% of controls reported a positive history of chickenpox. Overall, a positive history of chickenpox was associated with a 21% lower glioma risk, controlling for age and sex (Fig. 1A; mOR: 0.79, 95% CI: 0.65–0.96). A significant adverse OR was not observed at any site, and most site‐specific ORs were in the protective direction, though many did not reach statistical significance (possibly due to small numbers/inadequate statistical power). Two sites, Case Western Reserve University and Brigham and Women's Hospital, were excluded due to having cell counts below five.

Restricting to high‐grade gliomas, the mOR was slightly stronger and remained statistically significant (Fig. 1B; mOR: 0.74, 95% CI: 0.58–0.96), whereas among lower grade gliomas, the effect was attenuated and no longer statistically significant (Fig. 1C; mOR: 0.83, 95% CI: 0.66–1.05). Further stratifying by glioma diagnosis/study enrollment age group, we found that the strongest inverse association of chickenpox with high‐grade glioma risk was observed among the youngest age group (pOR: 0.53, 95% CI: 0.31–0.90, among participants <40 years of age at diagnosis/enrollment) [data not shown]. While the pORs among the older age groups remained similar to the overall estimate for high‐grade glioma risk (pOR: 0.81 and pOR: 0.79, for 40–59 and >59 years of age, respectively), they did not attain statistical significance. No patterns were observed by glioma diagnosis/study enrollment age for lower grade glioma risk, and none of the age‐stratified pORs were statistically significant.

The age at which participants developed chickenpox was also considered in our analyses [not shown]. A positive history of chickenpox was associated with an approximately 20–30% lower glioma risk, regardless of whether the participants developed chickenpox under age six (mOR: 0.70, 95% CI: 0.55–0.89), between the ages of six and nine (mOR: 0.74, 95% CI: 0.59–0.93), or above age nine (mOR: 0.76, 95% CI: 0.59–0.98).

In the overall study population, 10.3% of cases and 9.2% of controls reported having at least one episode of shingles. About 28% of participants reported having their first episodes of shingles before age 30 (overall median age: 44; median among cases: 44; median among controls: 43). A positive history of shingles was not significantly associated with glioma risk (mOR: 1.11, 95% CI: 0.89–1.38). The mORs were similar stratified by tumor grade.

## Discussion

In our study, a positive history of chickenpox was associated with a 21% lower glioma risk, adjusting for age and sex. The protective effect of chickenpox was stronger for high‐grade glioma, particularly among those under age 40. Our findings, which represent the results of the largest study to date on this topic, confirm the inverse associations previously reported in the literature on VZV and glioma.

The majority of published studies on VZV infection and glioma risk are from the San Francisco Bay Area Adult Glioma Study (SFBAGS) series 8, 9, 10, 11, 13. Using both self‐reported and serologic (anti‐VZV IgG) data to assess history of chickenpox, findings from this series have indicated that prior exposure to VZV is associated with an approximately 40% lower glioma risk 9, 10, 11. Although the odds ratio presented here is not quite as strong as those reported from the SFBAGS series, our estimate is based a larger study population and may possibly be more precise. However, additional studies, including meta‐analyses of all published findings, are necessary to estimate the true magnitude of effect.

Like our study, the SFBAGS analyses have implied that the inverse association with prior VZV infection may be stronger for high‐grade glioma 9, 11. For example, Wrensch et al. reported an OR of 0.6 for the association between anti‐VZV IgG positivity and any glioma (95% CI: 0.3–1.3), whereas their effect estimate when restricting to glioblastoma was stronger and attained statistical significance (OR: 0.4, 95% CI: 0.1–0.9) 11. Additionally, in a follow‐up study, the SFBAGS investigators observed that mean log anti‐VZV IgG levels were higher for controls than glioma cases, but were actually lowest for glioblastoma cases 9.

Besides the SFBAGS series, a few other epidemiologic studies have found similar associations between chickenpox and glioma risk 3, 12. Sjostrom et al. utilized specimens from three Scandinavian cohorts to investigate the association between VZV antibodies and glioma risk 12. Again, lower levels of anti‐VZV IgG were more common in glioma cases than in controls, particularly 2 years before diagnosis (OR: 0.63; 95% CI: 0.37–1.08; inter‐quartile *P* for trend = 0.03). Because of the use of prediagnostic specimens, this study provided further evidence that VZV antibody‐glioma associations reported in the literature are unlikely to be a result of postdiagnostic or treatment‐related factors (e.g., steroid use). Furthermore, such serologic studies also suggest that the associations observed between self‐reported history of chickenpox and glioma risk are unlikely to be completely attributable to memory problems or cognitive deficits in glioma patients.

A particularly interesting finding of our study is that the protective effect of chickenpox against high‐grade glioma was strongest among the youngest (<40) age group. Median age at glioma development is 55 years 16. It is possible that high‐grade gliomas that develop in younger individuals are etiologically heterogeneous from those that develop in older individuals. In fact, recent evidence indicates that potentially etiologically distinct glioma subtypes (defined by specific tumor molecular markers) have different ages at presentation 17. Nevertheless, our finding needs to be confirmed in other studies before definitive conclusions can be drawn, especially given that this observation was made among the smallest sample size of the three age groups examined.

In our study, shingles was not associated with glioma risk. Some previous studies have found an inverse association with glioma risk, though shingles has not been studied as frequently as chickenpox and has often been combined with chickenpox, rather than examined separately 9. In our study, the age at first shingles episode was skewed toward a younger distribution, compared to previous reports 18, 19, 20. In the U.S. and Europe, median age for shingles has been reported to be between 60 and 70 years. The median age in the GICC data was much younger (44 years). Although the incidence of shingles at younger ages may be increasing 21, 22, we believe that the age distribution reflected in our study is unlikely to be completely accurate. It is possible that some individuals are unsure of what shingles is or believed it to be synonymous with chickenpox or another viral rash. Unfortunately, there is no way to verify these data, and thus we must interpret our results on shingles cautiously.

The biological mechanism through which chickenpox may confer protection against glioma is currently unclear. One proposed mechanism is that VZV antibodies may demonstrate some cross reactivity to tumor cells (or other oncogenic viruses), and are thus capable of helping mount a protective immune response against existing tumor cells 9. Conversely, it is also possible that individuals who are more likely to develop cancer may be unable to mount strong immune responses to infections such as VZV 23.

A limitation of our study is the amount of intersite heterogeneity between our 14 international sites. Accordingly, we have provided site‐specific odds ratios and have used random‐effects meta‐regression in an effort to account for some of this heterogeneity. Because questionnaire administration methods and control types differed between sites, we have also conducted a number of sensitivity analyses (methods described in reference 15) to ensure that these differences did not detectably bias the results of our analyses.

Findings from the previous literature, bolstered by those of our study, provide strong epidemiologic rationale for continued investigation of the potential role of chickenpox (or other manifestations of VZV infection) in glioma development 3, 8, 9, 10, 11, 12, 13. Future studies will need to account for the potential impact of the VZV vaccine, which was licensed in 1995 in the U.S. for use among children 2 (and therefore cannot be evaluated in the older population of the GICC). Prior serologic analyses have demonstrated that antibody composition differs between children who experience a wild‐type VZV infection versus those who were received the vaccine 24. Some evidence indicates that antibodies against specific VZV‐encoded proteins (i.e., VZV ORF2 and IE63) may be more important than others in conferring protection against glioma 13, but the vaccine does not contain antigens corresponding to all 70 VZV open reading frames 1, 3, 24. Thus, future research ascertaining whether the vaccine confers similar protection against glioma as the wild‐type VZV infection is of high importance and may lend insight into the biological mechanisms at play.

## Conflict of Interest

The authors declare no conflicts of interest.
